# Directly Estimating Endmembers for Compressive Hyperspectral Images

**DOI:** 10.3390/s150409305

**Published:** 2015-04-21

**Authors:** Hongwei Xu, Ning Fu, Liyan Qiao, Xiyuan Peng

**Affiliations:** Depart of Automatic Test and Control, Harbin Institute of Technology, Harbin 150080, China; E-Mails: winaaa@163.com (H.X.); qiaoliyan@163.com (L.Q); pxy@hit.edu.cn (X.P.)

**Keywords:** hyperspectral images, distributed compressive sensing, endmember estimation, measurement matrix

## Abstract

The large volume of hyperspectral images (HSI) generated creates huge challenges for transmission and storage, making data compression more and more important. Compressive Sensing (CS) is an effective data compression technology that shows that when a signal is sparse in some basis, only a small number of measurements are needed for exact signal recovery. Distributed CS (DCS) takes advantage of both intra- and inter- signal correlations to reduce the number of measurements needed for multichannel-signal recovery. HSI can be observed by the DCS framework to reduce the volume of data significantly. The traditional method for estimating endmembers (spectral information) first recovers the images from the compressive HSI and then estimates endmembers via the recovered images. The recovery step takes considerable time and introduces errors into the estimation step. In this paper, we propose a novel method, by designing a type of coherent measurement matrix, to estimate endmembers directly from the compressively observed HSI data via convex geometry (CG) approaches without recovering the images. Numerical simulations show that the proposed method outperforms the traditional method with better estimation speed and better (or comparable) accuracy in both noisy and noiseless cases.

## 1. Introduction

Hyperspectral images (HSI) are collections of hundreds of images that have been acquired simultaneously in narrow and adjacent spectral bands, typically by airborne sensors [1,2]. Through the continued development of sensing technology, the spectral and spatial resolution for HSI has increased significantly. For example, the NASA Jet Propulsion Laboratory’s Airborne Visible Infra-Red Imaging Spectrometer (AVIRSI) covers the wavelength region from 0.4~2.5 microns using 244 spectral channels at the nominal spectral resolution of 10 nm [3]; the spatial resolution of the hyperspectral imager in the Tiangong 1 aircraft is 5 m [4]. High spectral and spatial resolution results in HSI providing a wealth of information for accurate target detection and identification, leading to many applications including environmental monitoring, agriculture planning, and mineral exploration [5]. However, it also makes the volume of data very large, which introduces a significant challenge to data transmission, storage and analysis. Due to the extremely large volume of HSI data, compression technology has received considerable interest in recent years.

In conventional HSI sensing systems, the full data are acquired and are then compressed before transmission. This paradigm has several disadvantages: first, all the data should be stored; second, the computationally costly implementation of the compression is required to reside on board, housed within the sensing modality. Typically, the sensor platform is a severely resource-constrained environment such as a plane or satellite. As an alternative to the conventional sensing systems, compressive sensing (CS) [6,7] is an effective approach to acquire and compress the data in only one step. CS theory shows that only a small collection of a sparse or compressible signal contains enough information for stable signal recovery. Distributed CS (DCS) extends the single signal CS to multiple signals [8,9,10]. By exploiting both intra- and inter-signal correlation structures, DCS can reduce the number of measurements of each signal effectively, saving on the costs of data storage, communication and processing. DCS is very suitable for multi-channel applications, such as HSI.

Blind hyperspectral unmixing (HU) is one of the most prominent research topics in signal processing (SP) for hyperspectral remote sensing [11,12]. Blind HU aims to identify endmembers present in a captured scene, as well as their proportions [13]. There are many methods for blind HU such as pixel purity index (PPI) [14], N-FINDR [15], vertex component analysis (VCA) [16], SSCBSS [17], hypGMCA [18], and modified VCA (MVCA) [19], which are all based on the Nyquist sampling theorem. There are also some HU methods based on the CS theory, such as CSU [20] and the method proposed in [5], but they all assume that the endmembers are known as side information. Endmember estimation is a key step to identify the materials in HSI, and in many applications, the endmembers are unknown.

The traditional method for endmember estimation under the CS/DCS framework consists of 2 steps: (1) recovering the HSI data by CS/DCS methods and (2) estimating the endmembers from the recovered data by HU methods. The recovery step takes considerable time and also introduces errors into the estimation step, which will degrade the speed and accuracy of the endmember estimation.

In this paper, by designing a type of coherent measurement matrix, we propose a novel method that estimates the endmembers directly from the compressive HSI with convex geometry (CG) approaches, which outperforms the traditional method with better estimation speed and better (or comparable) accuracy.

The paper is structured as follows. The necessary theoretical background and notations are provided in Section 2. In Section 3, we describe the proposed method in detail. The performance of the proposed method is demonstrated in Section 4 in comparison to the traditional method. We conclude the paper in Section 5. Important acronyms used in this paper are listed in Table 1.

**Table 1:** Important acronyms used in this paper.

Acronym	Meanings
HSI	Hyperspectral Images
SP	Signal Processing
CS	Compressive Sensing
DCS	Distributed Compressive Sensing
HU	Hyperspectral Unmixing
LMM	Linear Mixing Model
CG	Convex Geometry
JSM	Joint Sparse Model

## 2. Hyperspectral Unmixing in Distributed Compressive Sensing

### 2.1. Hyperspectral Unmixing

#### 2.1.1. Linear Mixing Model for HSI

**Figure 1:**
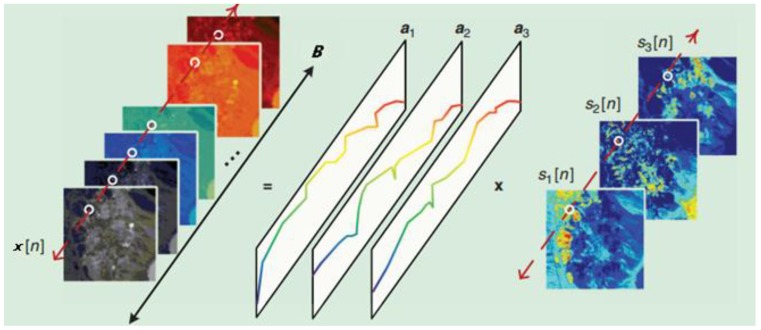
The linear mixing model of hyperspectral images [13].

HU refers to any process that separates the pixel spectral from a hyperspectral image into a collection of constituent spectral or spectral signatures, called endmembers and a set of fractional abundances, one set per pixel [12]. Mixing models can be characterized as either linear or nonlinear [12,13]. The linear mixing model (LMM) is a very representative model for HSI, and it is an acceptable approximation of the light scattering mechanisms in many real scenarios. In this paper, we only focus on the LMM. Let $x_{b}[n]$ denote the hyperspectral sensor’s measurement at spectral band b and at pixel  n. Let $\;x\left[n\right]=\left[x_{1}\left[n\right],x_{2}\left[n\right],\cdots ,x_{B}\left[n\right]\;\right]\;\epsilon \;R^{B}$, where B  is the number of spectral bands. The LMM can be denoted as

(1) $$x\left[n\right]=\overset{P}{\underset{i=1}{\sum }}s_{i}\left[n\right]a_{i}=s\left[n\right]A$$

for  n=1, 2,⋯,N, where each row vector $a_{i}\;\epsilon \;R^{B},\;i=1,\;2,\cdots ,P,$ is called an endmember signature vector, which contains the spectral information of a certain material (indexed by i). P is the number of endmembers, or materials; $A={\left[a_{1}^{T},\;a_{2}^{T},\cdots ,a_{P}^{T}\right]}^{T}\in R^{P\times B}$ is called the endmember matrix; and $s_{i}\left[n\right]$ is the proportion of endmember i at pixel n. $\;s\left[n\right]=\left[s_{1}\left[n\right],s_{2}\left[n\right],\cdots ,s_{P}\left[n\right]\;\right]\;\epsilon \;R^{P}\;$ is the proportion vector at pixel  n. N is the number of pixels. The LMM is shown in Figure 1.

Owing to physical constraints, the proportions are non-negative and satisfy the full additivity constraint:

(2) s[n]≥0, s[n]·1=1, n=1, 2,⋯,N

where 1 denotes an P×1 vector of ones;  s[n] ≥0 means that each entry in vector s[n] is non-negative.

Let $\;s_{i}={\left[s_{i}\left[1\right],s_{i}\left[2\right],\cdots ,s_{i}\left[N\right]\;\right]}^{T}\;\epsilon \;R^{N}\;$ denote the proportion vector of endmember  i, and let $x_{b}={\left[x_{b}\left[1\right],x_{b}\left[2\right],\cdots ,x_{b}\left[N\right]\;\right]}^{T}\epsilon \;R^{N}$ denote the measurements at band b. Let $\;S=\left[s_{1},s_{2},\cdots ,s_{P}\right]\;\epsilon \;R^{N\times P}$, and $\;X=\left[x_{1},x_{2},\cdots ,x_{B}\right]\;\epsilon \;R^{N\times B}$; then, the LMM can be written in the matrix form:

(3) X=SA

Obviously, row n  of X is  x[n], and row n  of S is  s[n]. From the signal processing aspect, S can be seen as the source matrix whose nth column contains the proportion of source n at each pixel; A is the mixing matrix, and X is the mixture matrix.

#### 2.1.2. Convex Geometry Approaches for HU

There are several key approaches for HU, including convex geometry (CG) approaches, statistical approaches, sparse regression approaches and nonnegative matrix factorization [12,13]. CG approaches are very popular and effective in HU. A vast majority of HU developments, if not all, are directly or intuitively related to concepts introduced in CG studies [13]. In this paper, we only focus on the CG approaches.

We introduce some mathematical notations in convex analysis: spanned space, affine hull and convex hull.

The space spanned by a set of vector $\left\{b_{1},\cdots ,b_{P}\right\}\;\epsilon \;R^{B}$ is defined as:

(4) $$\text{span}\left\{b_{1},\cdots ,b_{P}\right\}=\left\{\text{γ}=\sum_{i=1}^{P}{\text{β}}_{i}b_{i}{\text{|β}}_{i}\in R\right\}$$

The affine hull of a set of vectors $\;\left\{b_{1},\cdots ,b_{P}\right\}\;\epsilon \;R^{B}$ is the set of all affine combinations of elements of $\left\{b_{1},\cdots ,b_{P}\right\}$:

(5) $$\text{aff}\left\{b_{1},\cdots ,b_{P}\right\}=\left\{\text{γ}=\sum_{i=1}^{P}{\text{β}}_{i}b_{i}{\text{|β}}_{i}\in R,\;\sum_{i=1}^{P}{\text{β}}_{i}=1\right\}$$

The convex hull of a set of vector $\left\{b_{1},\cdots ,b_{P}\right\}\;\epsilon \;R^{B}$ is defined as:

(6) $$\text{conv}\left\{b_{1},\cdots ,b_{P}\right\}=\left\{\text{γ}=\sum_{i=1}^{P}{\text{β}}_{i}b_{i}{\text{|β}}_{i}\in R,{\text{β}}_{i}\geq 0,\;\sum_{i=1}^{P}{\text{β}}_{i}=1\right\}$$

Assuming that $\left\{b_{1},\cdots ,b_{P}\right\}$ are affinely independent, *i.e.*, $b_{2}-b_{1}$, $b_{3}-b_{1}$, …, $b_{P}-b_{1}$ are linearly independent, the convex hull of $\left\{b_{1},\cdots ,b_{P}\right\}$ is a (*P* − 1)-simplex in $R^{B}$.

**Figure 2:**
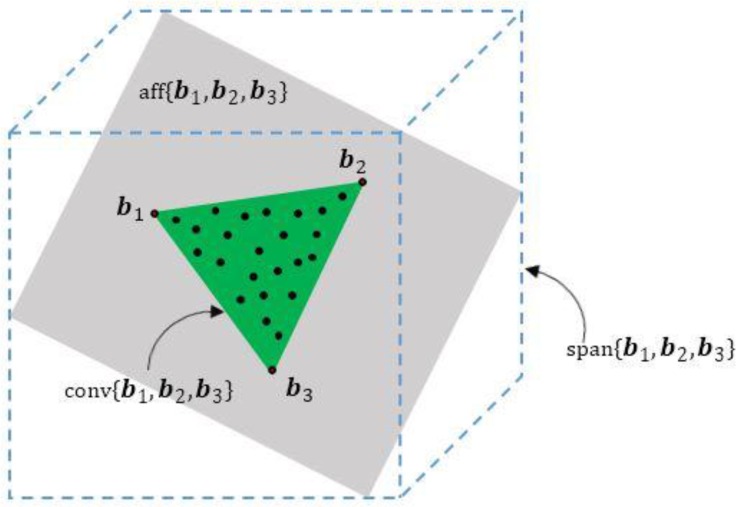
Affine hull and convex hull.

Figure 2 shows the illustration of the affine hull and convex hull for the case of  P=3. As can be seen, $\text{aff}\left\{b_{1},b_{2},b_{3}\right\}$ is a plane and $\text{conv}\left\{b_{1},b_{2},b_{3}\right\}$ is a triangle. $\left\{b_{1},b_{2},b_{3}\right\}\;$ are the vertices of the 2-simplex.

There is a strong connection between the convex hull and the LMM of HSI. From Equations (1)–(3), we can see that each measured hyperspectral pixel vector x[n] is a convex combination of the endmember $\;a_{1},\cdots ,a_{P}$:

(7) $$x\left[n\right]\in \text{conv}\left\{a_{1},\cdots ,a_{P}\right\},\;n=1,2,\cdots ,N$$

$\text{conv}\left\{a_{1},\cdots ,a_{P}\right\}$ is a simplex because $\left\{a_{1},\cdots ,a_{P}\right\}$ are linearly independent (and thus affine independent). Note that the vertices of $\text{conv}\left\{a_{1},\cdots ,a_{P}\right\}$ are $a_{1},\cdots ,a_{P}$; thus, in CG approaches, the inference of the endmember matrix A is equivalent to identifying the vertices of the simplex.

### 2.2. Compressive Sensing

CS theory indicates that if a signal is sparse or compressive in some basis, it can be exactly recovered by a small number of measurements, much less than the number required by the Nyquist sampling theory. Let $x=\;{\left[x\left[1\right],x\left[2\right],\cdots ,x\left[N\right]\right]}^{T}\;\epsilon \;R^{N}$ be a K sparse signal  (K≪N). The sparse basis is $\;\text{Ψ}\;\epsilon \;R^{N\times N}$ with a sparse coefficient vector $\;\eta \;\epsilon \;R^{N}$. The signal can be denoted as

(8) x=Ψη

with $\;||\eta {||}_{0}=K$, where $\;||\eta {||}_{0}=K$ denotes the number of non-zero entries in  η.

Φ  is the M×N measurement matrix, where M<N. The observation vector  y  consisits of M linear projections of  x:

(9) y=Φx=ΦΨη=Θη

where Θ=ΦΨ is called the sensing matrix.

**Figure 3:**
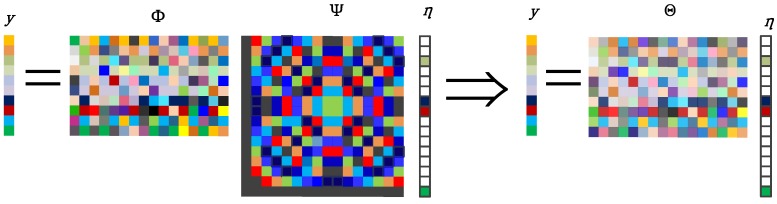
The framework of compressive sensing [22].

The design of Φ is critical for CS. A sufficient condition for stable signal recovery is that ΦΨ satisfies the RIP (Restricted Isometry Property) [21]. For each integer U=1, 2,⋯, define the isometry constant ${\text{δ}}_{U}$ of the matrix Θ as the smallest number such that $\left(1-{\text{δ}}_{U}\right)\text{||}\text{Θ}\eta {\text{||}}_{2}^{2}\leq \text{||}\text{Θ}\eta {\text{||}}_{2}^{2}\leq \left(1+{\text{δ}}_{U}\right)\text{||}\text{Θ}\eta {\text{||}}_{2}^{2}$ holds for all U-sparse vectors  η. We will loosely say that the matrix Θ  obeys the RIP of order U  if ${\text{δ}}_{U}$ is not too close to one [7]. The correlation between y and η is shown in Figure 3.

The length of y is much smaller than the length of  x, and thus, Equation (9) is underdetermined and the solution is ill-posed [23], for the matrix  Θ has more columns than rows. The most original approach for solving this problem is to find the sparsest vector  η, which seeks a solution to the $\;l_{0}$ minimization problem

(10) $$\text{min||}\eta {\text{||}}_{0}\;s.t.\;y=\text{Θ}\eta$$

The $\;l_{0}$ minimization is NP-hard and computationally intractable [23].

Fortunately, it has been proven that the $l_{1}$ minimization method can also exactly recover the signal under some conditions [7,21]. The $l_{1}$ minimization is given by:

(11) $$\text{min||}\eta {\text{||}}_{1}\;s.t.\;y=\text{Θ}\eta$$

The $l_{1}$ minimization is also called the basis pursuit (BP), whose computational complexity is $O(M^{2}N^{3/2})$ [24]. The recovery speed is very slow, especially when N is very large in the HSI application.

Greedy algorithms, such as Orthogonal Matching Pursuit (OMP) [25] and Subspace Pursuit (SP) [26], are more computationally attractable and are widely used for CS problems at the expense of requiring slightly more measurements [25,26,27].

### 2.3. Distributed Compressive Sensing

DCS [8,9,10] is a combination of distributed source coding (DSC) and CS. In the DCS framework, multichannel sensors measure signals that are each individually sparse in some domain and also correlated from sensor to sensor. The DCS theory rests on a concept called the joint sparsity of a signal ensemble. There are three joint sparse models (JSM): JSM-1, JSM-2 and JSM-3. In JSM-1, all signals are sparse and have common sparse components, while each signal contains sparse innovation parts. In JSM-2, all signals share the same support set with different amplitudes. In JSM-3, all signals have non-sparse common parts and sparse innovations.

**Figure 4:**
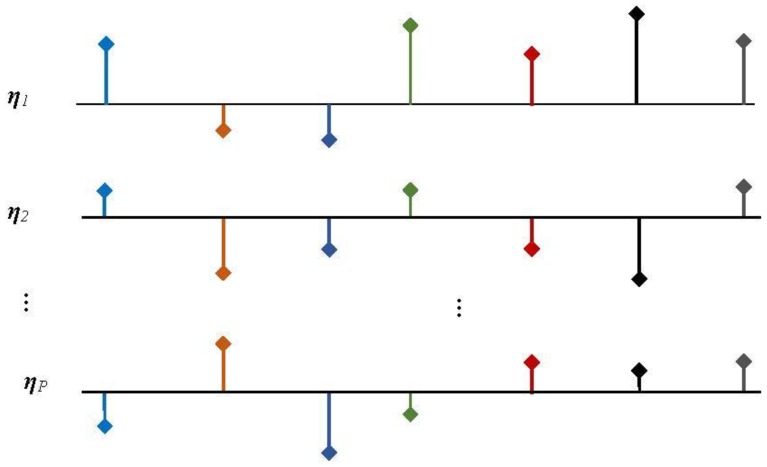
JSM-2 in distributed compressive sensing.

JSM-2 is the most concise model, as shown in Figure 4, and has been applied in compressive HSI [27].

A key prior that will be essential for compressive HSI is that each source image/mixture image is piecewise smooth in the spatial domain, implying a sparse representation in the DCT (discrete cosine transform) domain or the wavelet domain.

According to the assumption above, the image in each spectral band has a sparse representation, whose observations measured by the CS method can be written as

(12) $$y_{b}=\text{Φ}x_{b}=\text{ΦΨ}\eta_{b}=\text{Θ}\eta_{b}(b=1,2,\cdots ,B)$$

From our assumption; each source image is also sparse in some domain; and thus; the sparse representation of each mixture (spectral image) has the same sparse location with different coefficients due to the different mixing parameters of each source.

The SOMP (Simultaneous Orthogonal Matching Pursuit) method is proposed in [28] to reconstruct all of the signals that fall into the JSM-2 simultaneously, and this algorithm outperforms the OMP algorithm when dealing with multiple signals [8] and in compressively sensed HSI reconstruction applications [27].

Hence, the compressive observation of HSI can be denoted as

(13) $$Y=\left[y_{1},\;y_{2},\;\cdots ,y_{B}\right]=\left[\text{Φ}x_{1},\;\text{Φ}x_{2},\;\cdots ,\text{Φ}x_{B}\right]=\text{Φ}X=\text{Φ}SA$$

To summarize, the task of endmember estimation from compressive HSI can be described as follows: given the compressive observation matrix  Y, as well as the measurement matrix  Φ and the sparse basis  Ψ, estimate the endmember matrix  A.

### 2.4. Traditional Method for Endmember Estimation from Compressive HSI

**Figure 5:**
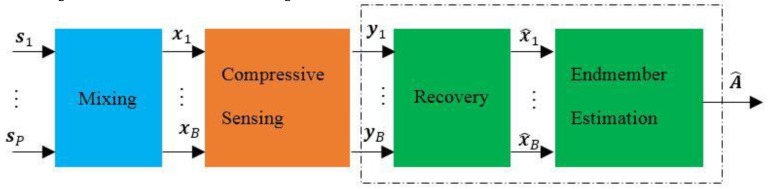
The framework of the traditional method based on DCS theory.

The framework of the traditional method for solving the problem mentioned above is shown in Figure 5. It contains two steps: in the first step, it recovers the hyperspectral image $x_{i}\;(i=1,\;2,\cdots ,B)$ by solving the DCS problem described in Equation (12) and then estimates the endmember matrix A (from the recovered mixtures) by solving the endmember estimation problem, as shown in Equation (3). In Figure 5, ${\hat{x}}_{b}\;$ is the recovered value of $\;x_{b}$, and $\hat{A}$ is the estimated value of  A.

The aim of endmember estimation is to estimate the endmember matrix  A, not the image matrix  X, which is only an intermediate representation used to calculate  A. However, the recovery of the images is a necessary step in the traditional method, as shown in Figure 5. It consumes a great deal of time and may also introduce errors to the estimation step.

In the next section, we will propose a new method that can estimate A directly from the compressive HSI data without recovering the images, which leads to a better estimation speed.

## 3. The Proposed Method

### 3.1. Framework Description of the Proposed Method

As discussed in Section 2, if we can estimate endmembers directly from the compressive HSI data without recovering the images, omitting the recovery step will greatly reduce the complexity of computation; we will obtain a much better estimation speed and possibly also a better estimation accuracy.

The compressive observation of HSI is denoted as follows:

(14) $$Y=\text{Φ}X=\text{Φ}SA=\left[\text{Φ}s_{1},\text{Φ}s_{2},\cdots ,\text{Φ}s_{B}\right]A=\left[v_{1},v_{2},\cdots ,v_{B}\right]A=VA$$

where $\;v_{p}=\;\text{Φ}s_{p}$ can be regarded as the compressive measurement of the source $\;s_{p}$.

The value $y_{b}=\text{Φ}x_{b}=\left(\sum_{p=1}^{P}\;v_{p}a_{pb}\right)\;$ is the compressive measurement of mixture $\;x_{b}$, and it is also the mixture of all of the $\;v_{p}(p=1,2,\;\cdots ,P)$. $a_{pb}$ is the element of matrix A at row p column b.

Equation (14) can be considered as LMM, as shown in Equation (3). Thus, we wish to estimate the endmember matrix  A directly via CG approaches such as PPI, N-FINDR, VCA, and MVCA.

Unfortunately, the properties of the measurement matrix Φ make it impossible to directly use the CG approaches on Equation (14). Incoherence is a critical property indicating that the structures of the measurement matrix used in CS that, unlike the signals of interest, have a dense representation in the basis  Ψ, and random matrices are largely incoherent with any fixed basis Ψ, making the sensing matrix  Θ  hold RIP with overwhelming probability [7]. Hence, $\;v_{p}=\;\text{Φ}s_{p}$ is not sparse in basis  Ψ, and v[n] (similar to the definition of x[n] in Section 2.1) cannot hold the non-negative and full additivity constraints, as shown in Equation (2), due to its dense and random character. We can see that:

(15) $$y\left[n\right]\in C=\left\{\text{γ}=\sum_{i=1}^{P}v_{i}\left[n\right]a_{i}\text{|}v_{i}\left[n\right]\in R\right\},\;n=1,\;2,\cdots ,N$$

$v_{i}[n]$ is the element in matrix V at column i row n. From Equation (15), we can see that C is not a convex hull, and y[n] is not a convex combination of endmember signatures. Actually, C is the space spanned by $\left\{a_{1},\cdots ,a_{P}\right\}$ (see Figure 2 for the relationship between the spanned space and the convex hull), and y[n] is a point in the space. In this condition, the endmember signatures $\left\{a_{1},\cdots ,a_{P}\right\}$ are no longer vertices of a simplex, which means that we cannot use CG approaches to estimate the endmembers directly.

To the knowledge of the authors and the referenced materials, we have not found a measurement matrix that not only satisfies the incoherence (or RIP), but also makes $\;v_{p}=\;\text{Φ}s_{p}$ sparse in basis Ψ or  v[n] hold the non-negative and full additivity constraints. It seems impossible to estimate endmembers from compressive HSI data directly.

**Figure 6:**
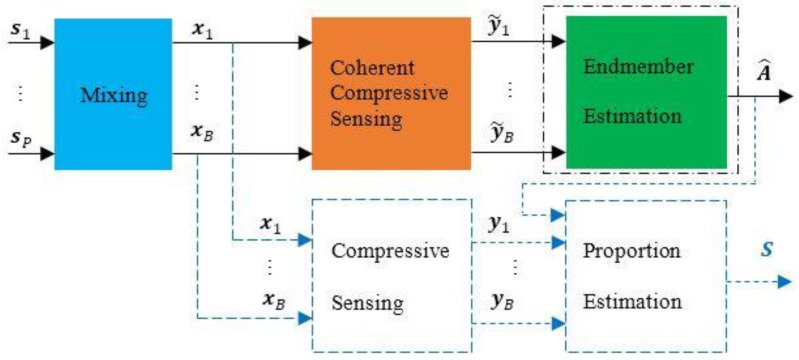
The framework of the proposed method.

We propose a novel method, as shown in Figure 6. A coherent measurement matrix is used to compressively measure the HSI, and the endmembers can be directly estimated from the observations.

First, we design a coherent measurement matrix that makes  v[n]  hold the non-negative and full additivity constraints.

The coherent matrix that does not hold the RIP cannot be used for exact and robust signal recovery, as mentioned above. In this paper, the aim is to estimate endmembers directly from the compressive HSI data, not to recover the HSI data. Therefore, we do not have to use an incoherent matrix (meaning, we do not have to use a random matrix). We give an example of such a coherent matrix. Let $\text{I}\;\epsilon \;R^{N\times N}$ be an identity matrix. We construct the measurement matrix using parts of  I. For example, we select one row in every  t(t=1, 2,…) rows from I to compose $\;\text{Φ}\;\epsilon {\text{ R}}^{M\times N}$ (the compression ratio is  t, $\;M=\text{round}\left(\frac{N}{t}\right)$, where round() is the operation of rounding towards the nearest integer). It is clear that v[n] holds the non-negative and full additivity constraints. With this kind of measurement matrix, it is possible to use CG approaches, such as PPI or VCA, to estimate the endmembers directly by solving the problem shown in Equation (14).

Other measurement matrices Φ  that make v[n] in  V=ΦS hold the non-negative and full additivity constraints can be used in this proposed method.

This type of measurement matrix achieves undersampling of HSI. It will lose some proportion information. Therefore, the undersampled data cannot be used to recover the proportion information. If this information is required, one can recover it by the data captured by an incoherent measurement matrix, as shown in Figure 6.

In this paper, we use the VCA algorithm to estimate the endmembers directly in the proposed method. We assume the presence of pure pixels in the undersampled data as required by VCA, and we also assume the presence of all of the materials in the data. These assumptions are easy to realize for the increasing spatial resolution of hyperspectral sensors.

In the proposed method, we first use a coherent measurement matrix to compressively sense HSI and then use the VCA method to estimate the endmember directly from the HSI observations without recovering the images, which is a necessary step in the traditional method. As shown in the dashed parts in Figure 6, if the proportion information S is required, one can use another incoherent measurement matrix to capture the global information of HSI, which can be used along with the estimated endmembers $\hat{A}$ to recover the proportions [5,20].

### 3.2. Analysis of the Computational and Memory Complexity

Under the linear observation model, HSI data are in a subspace of dimension  P. Typically,  P is far less than  B. The dimensionality B ranges from around 100 to 250, whereas P ranges from about 3 to 20 [29]. In the VCA algorithm, the HSI data dimensionality is reduced by PCA or SVD. The computational complexity of the proposed method is $\;O(P^{2}M$) [16].

The traditional methods used in this paper are SOMP-VCA (SOMP for HSI data recovery and VCA for endmember estimation) and OMP-MVCA. The computational complexity of the SOMP is  O(BKMN), where K is the sparsity of the signals. Typically, B and K are both larger than  P, so $\;BKMN>P^{2}MN\gg P^{2}M$. The computational complexity of VCA used in the traditional method is $\;O(P^{2}N$) (each spectral image length is N, while the length of the compressive spectral image in the proposed method is  M). The total computational complexity of the traditional method is $O(BKMN+P^{2}N$). In most cases, $\;BKMN>P^{2}MN\gg P^{2}N$, so the complexity can be simply denoted as  O(BKMN). The computational complexity of the SOMP-VCA is much larger than that of the proposed method. $P^{2}N>P^{2}M$, and thus, we can see that the computational complexity of the traditional method is larger than that of the proposed method, even if we use other CS recovery methods besides the SOMP algorithm. The computational complexity of OMP is O(BKMN) when K≪N, otherwise, the computational complexity is the larger one of $O\left(BK^{3}M\right)$ and O(BKMN). The computational complexity of MVCA is smaller than that of VCA. So the complexity of OMP-MVCA is dominated by OMP.

In the proposed method, the memory required is O(PM) due to dimensionality reduction and data compression. In the SOMP-VCA method, the memory required by SOMP is O(MN) and the memory required by VCA is  O(PN). MN≫PN>PM; therefore, the memory required by the SOMP-VCA method can simply be  O(MN), which is much larger than that of the proposed method. Similar to the analysis of SOMP-VCA, the memory required by OMP-MVCA is simply  O(MN).

## 4. Simulation Results

### 4.1. Evaluations of the Proposed Method

In this section, we first evaluate the practicability of the proposed method. Then, we compare the performance of the proposed method with the performance of the SOMP-VCA method.

**Figure 7:**
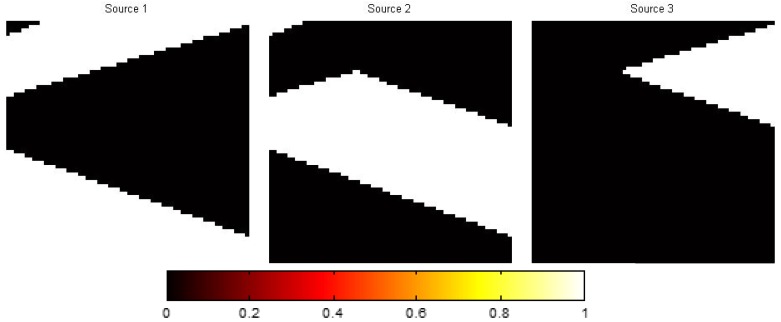
The proportion of each material in the rural HSI.

**Figure 8:**
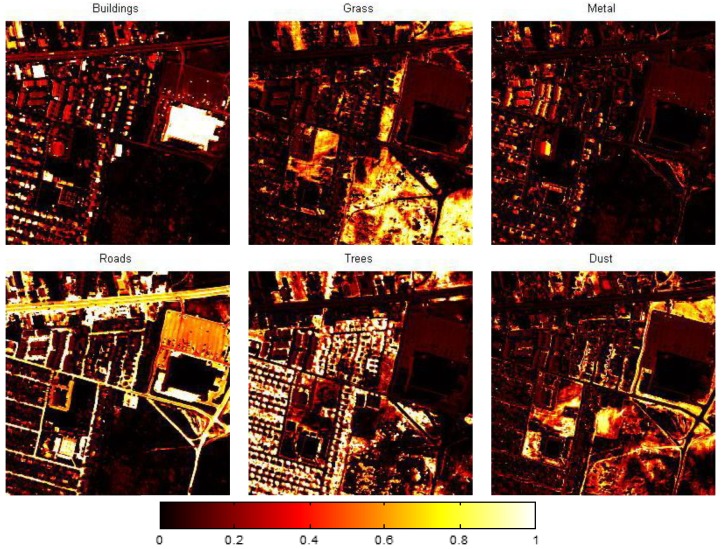
The proportion of each material in the urban HIS.

The data used in this paper are (1) the semi-synthetic HSI of a rural suburb of Geneva and (2) real-work urban HSI, both of which are also used in [5] (This dataset is available at [30]. We acknowledge Mohammad Golbabaee, Simon Arberet and Vandergheynst for providing the dataset.). In the rural HSI,  N=64×64, P=3, B=64, all the pixels are pure (which means that each pixel only contains one material), as shown in Figure 7. In the urban HSI,  N=256×256, P=6, B=171 (the first 16 bands are used in this paper), and parts of the pixels are pure, as shown in Figure 8. In this paper, the size of HSI data is reduced by SVD from N×B to N×P in VCA algorithm.

To evaluate the performance of the algorithms, we compute the rms (root-mean-square) error distance of vectors of angles $\;\text{θ}={\left[{\text{θ}}_{1},{\text{θ}}_{2},\cdots ,{\text{θ}}_{P}\right]}^{T}$ with

(16) $${\text{θ}}_{p}=\text{arccos}\frac{a_{p},{\hat{a}}_{p}}{\text{||}a_{p}\text{||}\cdot \text{||}{\hat{a}}_{p}\text{||}}\;,(p=1,\;2,\cdots ,P)$$

where ${\text{θ}}_{p}$ is the angle between vector $a_{p}$ and ${\hat{a}}_{p}$(the estimated value of $a_{p}$). Based on θ, we estimate the rms error distance:

(17) $$\text{rmsSAE }={\left(\frac{1}{P}\sum_{p=1}^{P}{\left({\text{θ}}_{p}\right)}^{2}\right)}^{1/2}$$

where *rmsSAE* measures the distance between $a_{p}$ and ${\hat{a}}_{p}$ for p=1, 2,⋯,P. (SAE stands for the Signature Angle Error).

We use $\tilde{\text{Φ}}$ to denote the coherent measurement matrix described in Section 3.1. In noisy cases, white Gaussian noise  n is added to the observation, *i.e.*, $\tilde{Y}=\tilde{\text{Φ}}X+n$. The SNR (signal-to-noise ratio) is from 20 to 40 dB with a step length of 10 dB. N is the number of pixels of a hyperspectral image, and M is the number of compressive measurements of an image. The compression ratio t  ($t=\text{round}(\frac{N}{M})$) is from 2 to 20 with a step length of 1 in the proposed method. We also consider the non-compression (Nyquist sampling) data, *i.e.*, t=1. Each experiment is repeated 50 times to calculate the mean result. The platform used in this paper is with the following hardware and software: (1) CPU: Intel Core i3 550, 3.2 GHz; (2) RAM: 4 GB; (3) OS: Windows 8, 64-bit; and (4) Matlab version: R2011b, 64-bit.

**Figure 9:**
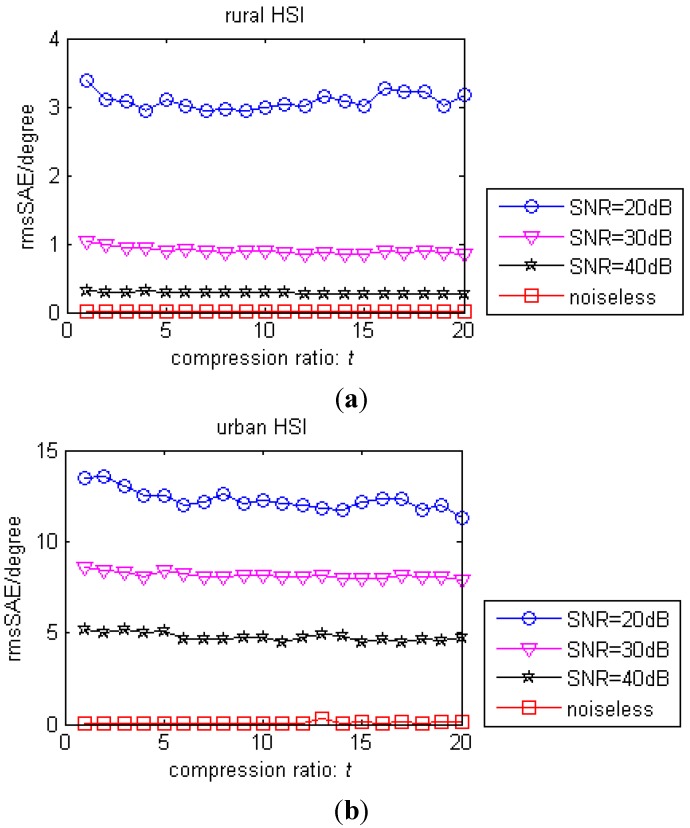
(**a**) Rural HSI: the rmsSAE of the proposed method as a function of the compression ratio *t* with different SNR; (**b**) Urban HSI: the rmsSAE of the proposed method as a function of the compression ratio *t* with different SNR.

**Figure 10:**
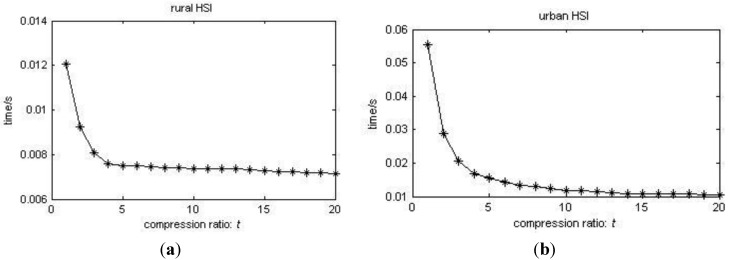
(**a**) Rural HSI: runtime of the proposed method as a function of compression ratio *t*; (**b**) Urban HSI: runtime of the proposed method as a function of compression ratio *t*.

From Figure 9 and Figure 10, we can see that as the compression ratio *t* grows, the rmsSAE does not change a lot, especially when the SNR is large, compared with the non-compression case *t* = 1 (this is due to the performance and the assumption of the VCA algorithm). However, the runtime decreases greatly as *t* increases compared with the non-compression case. We can see that the proposed method can estimate the endmember with comparable accuracy to the Nyquist-based method (*t* = 1) with much faster estimation speed as *t* increases, as long as the pure pixel and all material presence assumptions hold. We note that the value of *t* is user-defined as long as the presence of each material and pure pixel assumption holds.

To assess the performance of the proposed method for a large number of endmembers, we vary the number from P=10 to P=20. The endmember data are mineral signatures extracted from the U.S. Geological Survey (USGS) spectral library [16]. Each endmember signature consists of B=224 spectral bands. Each synthetic hyperspectral image has 4096 pixels.

**Figure 11:**
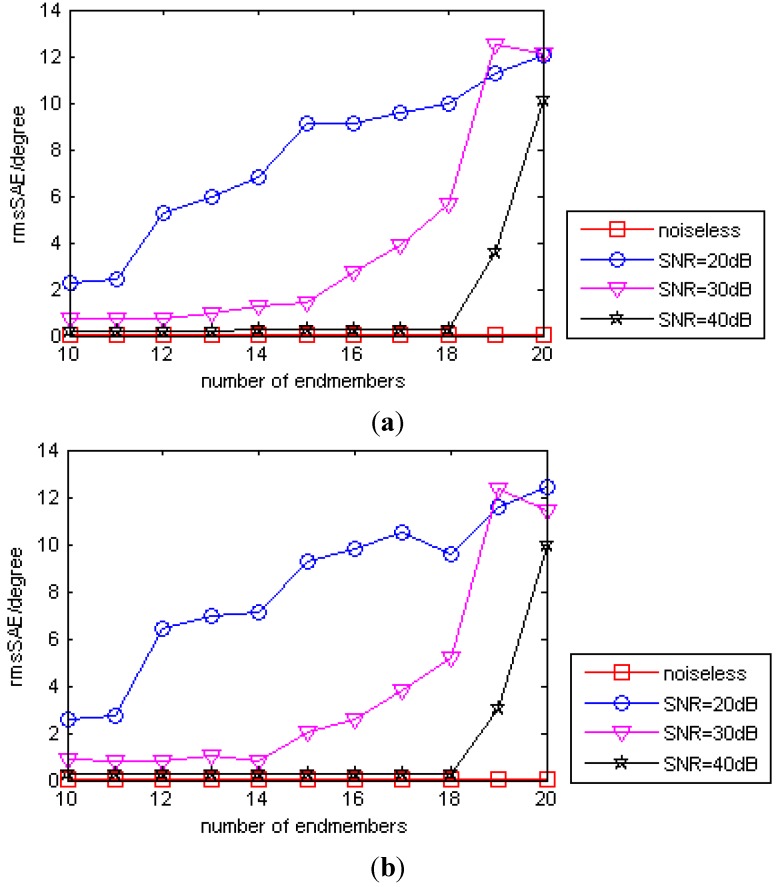
The rmsSAE of the proposed method as a function of the number of endmembers under different noise levels. (**a**) Compression ratio t = 10; (**b**) Compression ratio t = 20.

From Figure 11 we can see that the rmsSAE values increase roughly with the increase of the number of endmembers under the same noise level. And the rmsSAE values also decrease roughly with the increase of SNR, as expected. Comparing Figure 11a,b, we can also see that the performance of the proposed is comparabe when t=10 and t=20, as long as the pure pixel and all material presence assumptions hold.

### 4.2. Comparison of the Proposed Method and the SOMP-VCA Method

Next, we compare the performance of the proposed method with the traditional SOMP-VCA and OMP-MVCA methods.

In SOMP-VCA and OMP-MVCA methods, the compression ratio t ranges from 2 to 10 with a step length of 2. The incoherent measurement matrix ${\text{Φ}}_{T}$ is a random Gaussian matrix [21]. Each of the six signals (endmembers) in the urban HSI data has  N=65,536 points. They require too much memory, as described in Section 3.2, when processed by a computer (the computer used in the paper with 4 GB RAM cannot afford enough memory to run the SOMP-VCA/OMP-MVCA algorithm when t = 2 and 4). In the experiments below, we divide each signal into several segments, each of which consists of $N_{T}=4906$ points (The length of each signal in the rural HSI data is 4096). Therefore, ${\text{Φ}}_{T}$ is a $M_{T}\times N_{T}$ matrix, where $\;M_{T}=\text{round}(\frac{N_{T}}{t})$. To recover the HSI data by the traditional method, the sparsity K of each segment is required as *a priori* condition. We set $K=\text{round}(\frac{N_{T}}{100})$ in the experiments (Generally, it is very hard for people to get the exact value of K in practical applications. This is also a disadvantage of traditional methods). In noisy cases, the SNR is from to 20 to 40 dB with a step length of 10 dB. Each experiment was repeated 50 times.

From Table 2 and Table 3, we can see that at the same compression ratio, the rmsSAEs of the proposed method are much smaller than the rmsSAEs of both SOMP-VCA and OMP-MVCA methods when it is used for both the synthetic and real data. The reason is that both SOMP-VCA and OMP-MVCA methods first recover the images and then estimate endmembers from the recovered images, and the recovery step will introduce error to the estimation step. The proposed method directly estimates the endmembers by the VCA method without the recovery step. We can see that in the noiseless case, the proposed method can estimate the endmembers exactly, as some values of rmsSAE in Table 2 and Table 3 are 0.0000.

**Table 2:** Rural HSI: the rmsSAE of the proposed method, SOMP-VCA method and OMP-MVCA method under different noise levels.

SNR (dB)	Methods	Compression Ratio t				
		2	4	6	8	10
20	SOMP-VCA	7.2655	8.5649	11.8756	16.5145	22.4027
	OMP-MVCA	9.4577	10.4254	13.6821	17.6317	23.6413
	**proposed method**	**3.0957**	**2.9528**	**3.0154**	**2.9649**	**2.9956**
30	SOMP-VCA	7.2271	8.7619	11.1736	16.1245	21.8981
	OMP-MVCA	9.0371	10.8842	12.9093	17.5480	22.7639
	**proposed method**	**0.9858**	**0.9428**	**0.9147**	**0.8748**	**0.8937**
40	SOMP-VCA	7.4524	8.5810	11.4107	15.7571	21.5858
	OMP-MVCA	9.1606	10.4191	12.9728	16.7786	22.8731
	**proposed method**	**0.3022**	**0.3026**	**0.2853**	**0.2850**	**0.2823**
noiseless	SOMP-VCA	7.1363	8.7955	11.1507	15.8788	21.1658
	OMP-MVCA	8.7426	9.7065	12.4191	16.2971	22.1178
	**proposed method**	**0.0000**	**0.0000**	**0.0000**	**0.0000**	**0.0000**

**Table 3:** Urban HSI: the rmsSAE of the proposed method, SOMP-VCA method and OMP-MVCA method under different noise levels.

SNR (dB)	Methods	Compression Ratio t				
		2	4	6	8	10
20	SOMP-VCA	24.9187	31.6129	36.0830	36.6952	37.4615
	OMP-MVCA	32.3194	34.4224	38.1644	41.1802	42.9744
	**proposed method**	**13.5762**	**12.5006**	**11.9720**	**12.5650**	**12.2655**
30	SOMP-VCA	26.1574	32.6052	35.9462	36.4788	38.6030
	OMP-MVCA	32.4168	34.6774	37.7206	41.1756	42.3880
	**proposed method**	**8.4225**	**8.0781**	**8.2451**	**8.0752**	**8.1576**
40	SOMP-VCA	25.2347	31.5503	35.5626	36.4574	37.4830
	OMP-MVCA	32.8362	35.3572	38.7706	41.4157	43.3261
	**proposed method**	**5.0213**	**5.0131**	**4.6558**	**4.6736**	**4.7675**
noiseless	SOMP-VCA	24.4760	32.2732	35.1763	37.2746	38.6559
	OMP-MVCA	31.7520	34.7726	38.8428	41.8539	43.3041
	**proposed method**	**0.0000**	**0.0658**	**0.0000**	**0.0634**	**0.0000**

Standard deviations of rmsSAE under different conditions are listed in Table 4 and Table 5. We can see that under the same condition, the standard deviation values of the proposed method are also smaller than the values of SOMP-VCA or OMP-MVCA methods. The results indicate that the convergence of the proposed method is the best among the three methods.

**Table 4:** Rural HSI: the standard deviations of rmsSAE of the proposed method, SOMP-VCA method and OMP-MVCA method under different noise levels.

SNR (dB)	Methods	Compression Ratio t				
		2	4	6	8	10
20	SOMP-VCA	0.8902	1.4799	1.7669	1.9100	2.4746
	OMP-MVCA	0.9173	1.4002	1.7810	1.9418	2.4016
	**proposed method**	**0.2902**	**0.3109**	**0.2836**	**0.3009**	**0.2839**
30	SOMP-VCA	1.1270	1.4565	1.8702	1.9582	2.1471
	OMP-MVCA	0.8975	1.4758	1.6597	1.8604	2.3451
	**proposed method**	**0.0925**	**0.0946**	**0.0875**	**0.0937**	**0.1071**
40	SOMP-VCA	0.9530	1.3130	1.7083	1.9289	2.4834
	OMP-MVCA	0.9719	1.9057	1.9287	2.4761	2.6789
	**proposed method**	**0.0291**	**0.0295**	**0.0322**	**0.0273**	**0.0344**
noiseless	SOMP-VCA	0.9990	1.4686	2.0272	2.2618	2.3057
	OMP-MVCA	0.9692	1.6301	2.0236	2.5843	2.4048
	**proposed method**	**0.0000**	**0.0000**	**0.0000**	**0.0000**	**0.0000**

**Table 5:** Urban HSI: the standard deviations of rmsSAE of the proposed method, SOMP-VCA method and OMP-MVCA method under different noise levels.

SNR (dB)	Methods	Compression Ratio t				
		2	4	6	8	10
20	SOMP-VCA	3.6738	5.7059	9.7719	9.6847	11.0516
	OMP-MVCA	5.8462	7.3156	10.5079	11.9029	12.2469
	**proposed method**	**2.4330**	**1.8586**	**2.0141**	**2.2015**	**1.8326**
30	SOMP-VCA	3.6221	5.7251	9.8847	9.8163	10.9784
	OMP-MVCA	5.8731	6.8789	10.5414	11.8779	12.2589
	**proposed method**	**0.6661**	**0.7820**	**0.7265**	**0.5924**	**0.8368**
40	SOMP-VCA	3.6785	5.8697	9.7896	9.8166	11.2482
	OMP-MVCA	5.7435	7.3800	10.6197	11.7198	12.6990
	**proposed method**	**1.4182**	**1.2347**	**1.2149**	**1.2545**	**1.4128**
noiseless	SOMP-VCA	3.5570	5.7007	9.8128	9.8786	11.2197
	OMP-MVCA	5.5244	7.5697	10.9623	11.8132	12.7545
	**proposed method**	**0.0000**	**0.0781**	**0.0000**	**0.0673**	**0.0000**

From Table 6 and Table 7, we can see that the time consumed by the proposed method is much smaller than the time consumed by SOMP-VCA or OMP-MVCA methods. As analyzed in Section 3, the proposed method estimates the endmembers in one step, while both SOMP-VCA and OMP-MVCA use two steps. Therefore, from Table 2 to Table 7, the estimation accuracy and speed of the proposed method are both better than those of SOMP-VCA and OMP-MVCA methods.

**Table 6:** Rural HSI: average runtime consumed by the two methods for estimating the endmembers.

Compression Ratio t	SOMP-VCA	OMP-MVCA	Proposed Method
2	21.1327	172.9077	0.0092
4	10.8779	96.5817	0.0076
6	7.4465	71.5078	0.0075
8	5.7839	58.4532	0.0075
10	4.7957	51.1818	0.0073

**Table 7:** Urban HSI: average runtime consumed by the two methods for estimating the endmembers.

Compression Ratio t	SOMP-VCA	OMP-MVCA	Proposed Method
2	113.5758	692.2542	0.0287
4	59.8402	385.9496	0.0168
6	41.8393	285.0992	0.0143
8	32.9574	233.9354	0.0130
10	27.7860	204.1461	0.0118

Different CS recovery algorithms affect the performance and the runtime for estimating the endmembers. To eliminate the influence of the choice of the CS method, we suppose that the sparsity of each signal is known and that the CS method can recover the HSI data accurately, and the recovered data are as accurate as the Nyquist-based data at the same noise level. From Figure 9 and Figure 10, we can see that the performance of the proposed method (t > 1) is comparable to the performance of the method with Nyquist-based data ( t  = 1). Therefore, the performance of the proposed method is comparable to or better than the performance of the traditional CS base method. The same is true of the runtime time of the proposed method: it is less than that of the traditional CS base method with other CS recovery methods, as analyzed in Section 3.2.

## 5. Conclusions

In this paper, we proposed a new method to directly estimate the endmembers from the compressive observations of the HSI data, while traditional methods first have to recover the HSI data from the compressive observations and then estimate the endmembers. Simulation results demonstrated that the proposed method outperforms the traditional method with better estimation speed and better (or comparable) accuracy.
